# Diseases of the Fingers : A.—The Nails

**Published:** 1891-08-01

**Authors:** 


### SURGERY.

### DISEASES OF THE FINGERS.

#### A.—The Nails.

The affections to which the fingers are liable owe their peculiarities to the anatomical structure of the parts involved; and all treatment of a local nature must depend for its success on an appreciation of this structure.

We have, firstly, the phalangeal bones, with their vascular epiphyses at the proximal ends. It is to be noted that union to the shaft takes place as late as the twentieth year; this allows partial or complete separation to occur after blows and severe strains, but the position of the tendons and their strong insertions usually prevent much displacement, and the injury is easily overlooked. There is a strong periosteum, and this is firmly connected to the fibrous tissue which is so abundant, especially in the terminal phalanx. The irregular bundles of connective tissue form the so-called "pulp" of the fingers, and bind down the skin on all sides to the bone. In this tissue the blood vessels are large and well supported; hence the "pulsation" which is so often complained of in painful affections of this part.

Not only is the "dermis" particularly firm and unyielding, but the "epidermis" is modified on the back of the terminal phalanx to form the nail. This consists of the so-called *stratum lucidum* of the skin, with a small amount of the deeper layers and none of the most superficial, the *stratum corneum.*

Klein has investigated the development of the nail in the fœtus, and has found that to about the middle of the *fifth* month of intra-uterine life there is a distinct "horny" layer (*stratum corneum*) over the whole finger. By the *seventh* month this has disappeared, and the nail presents the shining smooth aspect characteristic of its healthy adult state.

It is quite obvious that the nail must, therefore, be considered as part of the "epidermis," modified. We should expect to find, therefore, very few diseases attack it, for the "epidermis" *alone* cannot be said to suffer much. Its diseased conditions depend on the vascular tissue lying beneath it for the most part, and this is the case with the nail. So-called "*onychia*" is generally an affection of the matrix or of the bed on and from which the nail grows. Modifications of these underlying parts of course affect the superficial nail secondarily; if the circulation in the veins is impeded, the peculiar shaped nail so often found in phthisis results; if the arterial circulation is feeble, as in acute illnesses, the growth of the nail is stopped for a time, and so on.

*Nervous disturbance* also affects the health of the nails. In *Multiple neuritis* they have been found to be rough and to have lost their polish, and Bielschowsky has recently described a case in which white patches occurred at the lower part of the finger nails in this disease.

In the malformations of the fingers which sometimes occur in psoriasis (supplement to *Medical Journal,* July 18th, 1891) the nails participate in the destructive changes.

The irregularities, overgrowths, deficiencies, &c., that are so common owe their origin to disease of the matrix, primarily, not of the nail itself.

This matrix is really the corium of the nail and is very firmly fixed to the periosteum. It is highly vascular, as is the bed on which the nail lies and from which it partially grows.

It is convenient to adopt the term *matrixitis* (Agnew, &c.), for diseases of this part.

*Matrixitis* or *Onychia* is a common affection. It usually occurs in the thumb when it arises from local causes; when of constitutional origin it is apt to attack several fingers. It is characterised by "pain, redness and swelling" at the root of the nail and this is soon followed by very tender ulceration, which spreads round the nail-bed. It is so frequently found in syphilitic and scrofulous people that it has been looked upon as a special manifestation of these diatheses. This is chiefly due, however, to the fact that the inheritors of these diseases (it is found in congenital rather than in acquired syphilis) are unhealthy and of low vitality; and their tissues are not in a condition to resist inflammation.

The treatment is local and constitutional; but it is not necessary in the supposed syphilitic cases to give mercury; tonics are indicated and the local treatment should be removal of the diseased nail in the bad cases and hot fomentations in the mild.

*In-growing* finger nails are rare; the pressure, which is so important a factor in the toes, is absent; but it often happens that a dead nail is allowed to remain *in situ,* and the consequences are that the sides of the vascular bed and the matrix itself are constantly irritated by this foreign body, and become inflamed. Granulations spring up, usually at the side of the nail, and a great deal of pain results.

Dirt of all kinds is a fruitful source of diseases of the matrix. This may be introduced at the sides of or beneath the nail, or it may make its entry by a prick or scratch. Matrixitis results, and suppuration often follows, leading occasionally to one form of whitlow. The germs of ringworm (tinea tonsurans) sometimes become grafted at the sides of the nail, and involve the nail itself in much the same manner as they do the hairs, causing splitting and disintegration. This is called Onychomycosis, and is a rather troublesome affection. It is necessary in its treatment to apply some alkaline wash to the parts first, in order to soften the nail, and then to rub in the germicide.

Some people suffer from a troublesome *longitudinal splitting* of the nails of the fingers. In such cases it is usually found possible to introduce a probe between the nail and the "bed," indicating a want of proper union between the two. This shows imperfect circulation and nutrition at the ends of the fingers, and is due more to constitutional than to local causes. Tight gloves and strongly alkaline soaps favour the production of this condition.

We thus see that the diseases of the nail itself are almost always *secondary*, that is, they depend on some abnormal condition of the underlying bed and matrix, usually the latter. Constitutional conditions, such as struma, syphilis, and acute illnesses powerfully affect the nails; also impediments in the circulation (as in phthisis, &c.) modify their growth. The matrix, on the other hand, is liable to several distinct diseases, and these are both local (due to pricks, introduction of dirt or germs, &c.) or constitutional. The anatomical conditions of the part make it necessary to treat these promptly and energetically, incisions to relieve tension and removal of the nail being often necessary.

